# Tobacco Smoking Increases Methylation of Polypyrimidine Tract Binding Protein 1 Promoter in Intracranial Aneurysms

**DOI:** 10.3389/fnagi.2021.688179

**Published:** 2021-07-06

**Authors:** Zhepei Wang, Shengjun Zhou, Jikuang Zhao, Sheng Nie, Jie Sun, Xiang Gao, Cameron Lenahan, Zhiqin Lin, Yi Huang, Gao Chen

**Affiliations:** ^1^Department of Neurosurgery, Ningbo Hospital, Zhejiang University School of Medicine, Ningbo, China; ^2^Department of Neurosurgery, The Second Affiliated Hospital, Zhejiang University School of Medicine, Hangzhou, China; ^3^Burrell College of Osteopathic Medicine, Las Cruces, NM, United States

**Keywords:** intracranal aneurysm, DNA Methylation, polypyrimidine tract binding protein 1, tobacco smoking, CpG island

## Abstract

DNA methylation at the gene promoter region is reportedly involved in the development of intracranial aneurysm (IA). This study aims to investigate the methylation levels of polypyrimidine tract-binding protein 1 (*PTBP1*) in IA, as well as its potential to predict IA. Forty-eight patients with IA and 48 age- and sex-matched healthy controls were recruited into this study. Methylation levels of CpG sites were determined via bisulfite pyrosequencing. The *PTBP1* levels in the blood were determined using a real-time quantitative reverse transcription-polymerase chain reaction test. Significant differences were found between IAs and controls in CpG1 (*p* = 0.001), CpG2 (*p* < 0.001), CpG3 (*p* = 0.037), CpG4 (*p* = 0.003), CpG5 (*p* = 0.006), CpG6 (*p* = 0.02), and mean methylation (*p* < 0.001). The mRNA level of *PTBP1* in the blood was much lower in IAs compared with controls (*p* = 0.002), and the *PTBP1* expression was significantly associated with DNA methylation promoter levels in individuals (*r* = −0.73, *p* < 0.0001). In addition, stratification analysis comparing smokers and non-smokers revealed that tobacco smokers had significantly higher levels of DNA methylation in *PTBP1* than non-smokers (*p* = 0.002). However, no statistical difference in *PTBP1* methylation was found between ruptured and unruptured IA groups (*p* > 0.05). The ROC analyses of curves revealed that *PTBP1* methylation may be a predictor of IA regardless of sex (both sexes, area under curve (AUC) = 0.78, *p* < 0.0001; male, AUC = 0.76, *p* = 0.002; female, AUC = 0.79, *p* < 0.0001). These findings suggest that long-term tobacco smoke exposure led to DNA methylation in the promoter region of the *PTBP1* gene, which further decreased *PTBP1* gene expression and participated in the pathogenesis of IA. The methylation of *PTBP1* may be a potential predictive marker for the occurrence of IA.

## Introduction

An intracranial aneurysm (IA) is a cystic pathological dilatation of the intracranial arteries. The walls of cerebral arteries are prone to rupture when they become too weak to resist hemodynamic pressure, leading to distention (Etminan et al., 2014). As a severe disease, IA has a prevalence and mortality of 7% and 30%, respectively, in ages 35–75 among the Chinese population (Chen et al., 2018). Previous studies have demonstrated that hemodynamic stress, vascular risk factors (i.e., hypertension, hyperlipidemia, arteriosclerosis, and smoking), and genetic susceptibility play an important role in the formation of IA (Kassam et al., 2004; Frosen et al., 2012, 2013). However, the mechanisms of IA pathogenesis are not fully understood.

DNA methylation is a common epigenetic modification that regulates gene expression by recruiting proteins involved in gene repression or by inhibiting the binding of transcription factors to DNA (Moore et al., 2013). The levels of DNA methylation can be altered by environmental shifts, such as a change in nutritional status or environmental exposures (e.g., smoking), which affects DNA conformation, stability, and its ability to interact with proteins (Tsaprouni et al., 2014). A previous study found that smoking can induce cytochrome-c oxidase subunit II aberrant methylation and apoptosis in human umbilical vascular endothelial cells (Yang et al., 2015). Tobacco smoking was a risk factor for cerebrovascular disease, including IA, which participated in the process of arterial vascular disease through epigenetic regulation of DNA methylation (Siemelink et al., 2018). Another study suggested that DNA methylation played an important role in the development of IA, involving the regulation of immune and inflammatory responses, cell functions, and cell signal transduction (Yu et al., 2017).

Polypyrimidine tract-binding protein (*PTBP1*), also known as hnRNP I, is a member of the heterogeneous nuclear ribonucleoprotein family, which has four RNA-binding domains, and plays a vital role in alternative splicing, mRNA stability, localization, and polyadenylation (Singh et al., 1995; Wollerton et al., 2004). Expression of *PTBP1* positively correlates with the growth of various cancers, such as brain tumors (Cheung et al., 2009), breast cancers (He et al., 2014), primary colorectal tumors (Takahashi et al., 2015), and various malignant cell lines (Wang et al., 2008), thus indicating poor prognosis. *PTBP1* is a main regulator of the enzyme, pyruvate kinase (PK), which plays an important role in the abnormal growth and enhanced glycolysis of pulmonary arterial endothelial cells (PAEC) in pulmonary hypertension (Caruso et al., 2017). Besides, it has also been proven that *PTBP1* is expressed in vascular smooth muscle cells (VSMCs) (Gooding et al., 2003), which are involved in regulating the expression of endothelial nitric oxide synthase (Yi et al., 2015), promoting proliferation and dedifferentiation of VSMC to cause neointimal hyperplasia (Kang et al., 2013; Wang et al., 2019). Therefore, we hypothesized that the *PTBP1* gene promotor of DNA methylation would be associated with the pathological development of IA.

In this study, we recruited 48 angiography-proven IA patients and 48 age- and sex-matched healthy individuals from Eastern China, and we performed a case-control test to validate the role of *PTBP1* gene promotor of DNA methylation in increasing the risk of IA in our homogenous samples.

## Materials and Methods

### Sample and Clinical Data

This research was approved by the Ethics Committee of Ningbo First Hospital. A total of 48 IA (48.08 ± 0.82 years) cases and 48 age-/sex- matched controls (46.63 ± 0.87 years) were recruited from the Ningbo First Hospital between September 2015 and December 2016. All of the patients with IA were diagnosed according to standard classifications using 320 dynamic state volume computed tomography angiography or digital subtraction angiography. All of the healthy control subjects were excluded from cardiovascular diseases, cerebrovascular diseases, liver and kidney diseases, as well as other serious diseases, such as malignant tumors.

Clinical data, including age, sex, and plasma levels of high-density lipoprotein (HDL), low-density lipoprotein (LDL), triglycerides (TG), and total cholesterol (TC) were collected for analysis. Blood samples were collected from participants on the first day of hospitalization. The levels of HDL, LDL, TG, and TC were measured using standard enzymatic methods, and were assessed using the automatic biochemical analyzers (Olympus AU2700, Japan).

### Pyrosequencing Assay

Human genomic DNA extraction was performed using an automatic nucleic acid extraction instrument (Lab-Aid 820, Xiamen City, China) using a magnetic bead isolation method. Six GpG dinucleotides on a fragment (chr19:796,392–797,191) within the promoter region of the *PTBP1* gene were selected for DNA methylated pyrosequencing. The EpiTect Bisulfite kit (Qiagen, Hilden, Germany) was used for the sodium bisulfite DNA conversion. DNA methylation was performed on a PyroMark System (Qiagen, Hilden, Germany). The polymerase chain reaction (PCR) primers were designed using the PyroMark Assay Design software (Qiagen, Hilden, Germany). The sequences of the PCR primers for the six CpG regions of the PBTP1 gene are listed as follows: the forward primer, 5′-GTATTTGTGGTTATTGTGGAAATAGT-3′; the reverse primer, 5′-Biotin-AAAACCCTCAAAACTCCATATTAATT-3′; the sequencing primer, 5′-GGTTATTGTGGAAATAGTT-3′.

### Quantitative Real-Time Polymerase Chain Reactions

The total RNA was extracted from the blood using TRIzol reagent (Invitrogen, CA, United States) according to the manufacturer’s detailed instructions. The quality of the RNA was assessed using a NanoDrop spectrophotometer (Thermo Fisher Scientific, MA, United States). Real-time quantitative reverse transcription−polymerase chain reaction (qRT−PCR) was performed on the LightCycler 480 system (Roche, Mannheim, Germany) using the SYBR Green Kit (TaKaRa, Dalian, China). The GAPDH mRNA was used as the reference for quantification. The primers and PCR application conditions for GAPDH (forward primer, 5′-CAACGGATTTGGTCGTATTGG-3′ reverse primer, 5′-TGATGGCAACAATATCCACTTTACC-3′) and *PTBP1* (forward primer, 5′-GCTCAGGATCATCGTGGAGAA-3′; reverse primer, 5′-ATCTTCAACACTGTGCCGAACTT-3′) were described in a previous study (Santiago and Potashkin, 2015).

### Statistical Analyses

The DNA methylation levels and the clinical data between different groups were presented as means ± standard error (SE) or number. The correlation between *PTBP1* methylation and clinical data was analyzed using Pearson’s correlation test. The normally distributed data were analyzed using the independent samples *t*-test, and the abnormally distributed data were analyzed via a non-parametric test. A receiver operating characteristic (ROC) curve was used to evaluate the sensitivity of the *PTBP1* methylation for the diagnosis of IA. SPSS software version 23.0 (IBM Corp. Armonk, NY, United States) was used for statistical analyses, and GraphPad Prism V8.0 (La Jolla, CA, United States) was used for the figure analysis. Power analysis was performed using Power and Sample Size Calculation software (Nashville, TN, United States). The data were considered statistically significant at *p* < 0.05.

## Results

**FIGURE 1:**
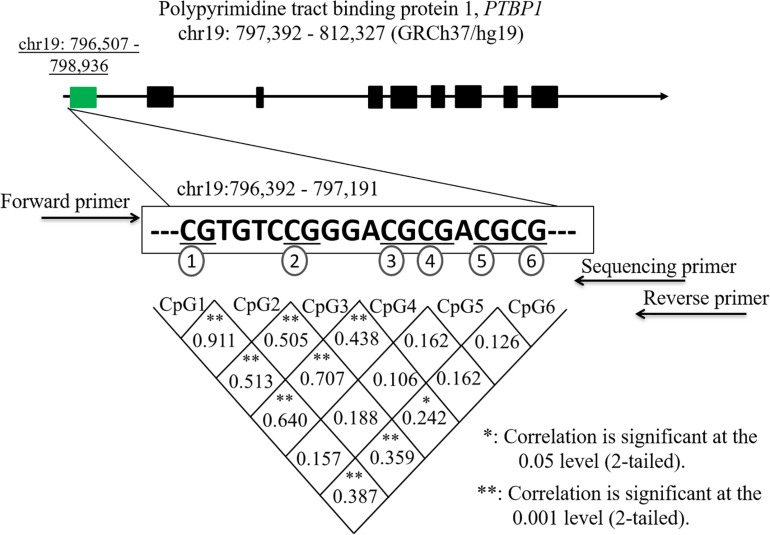
Correlations among six CpG sites in the *PTBP1* gene. *p < 0.05, **p < 0.001.

The clinical characteristics between IA patients and controls are shown in Table 1. No significant differences were observed between the two groups regarding clinical data, such as age, sex, hypertension, diabetes, drinking, smoking, TG, TC, HDL, and LDL (*p* > 0.05). Six CpG dinucleotides in the promoter region (hg19, chr19:796,392–797,191) of the *PTBP1* gene were selected to measure DNA methylation levels (Figure 1). Significant correlations were found among the levels of CpG1, CpG2, CpG3, CpG4, and CpG6 methylation (*p* < 0.05).

**TABLE 1:** Clinical characteristics of all participants.

Characteristics	Controls (*n* = 48)	IAs (*n* = 48)	t/x	*p*
Age (year)	46.63 ± 0.87	48.08 ± 0.82	1.22	0.226
Sex (Male, n)	24	24	0.03	0.859
Hypertension (n)	3	8	1.23	0.267
Diabetes (n)	4	2	0.14	0.710
Drinking (n)	6	10	0.43	0.512
Smoking (n)	10	13	0.11	0.742
TG (mmol/L)	1.57 ± 0.20	1.53 ± 0.12	0.18	0.856
TC (mmol/L)	4.77 ± 0.13	4.95 ± 0.11	0.99	0.321
HDL (mmol/L)	1.27 ± 0.04	1.17 ± 0.03	1.81	0.073
LDL (mmol/L)	2.91 ± 0.09	3.05 ± 0.09	1.01	0.317

As shown in Table 2, significant differences were found in CpG1 (IAs vs. Controls: 3.27 ± 0.26 vs. 1.89 ± 0.31, *p* = 0.001), CpG2 (IAs vs. Controls: 4.32 ± 0.28 vs. 2.59 ± 0.36, *p* < 0.001), CpG3 (IAs vs. Controls: 2.22 ± 0.21 vs. 1.20 ± 0.48, *p* = 0.037), CpG4 (IAs vs. Controls: 1.72 ± 0.24 vs. 0.74 ± 0.22, *p* = 0.003), CpG5 (IAs vs. Controls: 1.94 ± 0.59 vs. 0.22 ± 0.16, *p* = 0.006), and CpG6 (IAs vs. Controls: 1.37 ± 0.40 vs. 0.32 ± 0.18, *p* = 0.02). Therefore, the mean DNA methylation level in IA patients (2.48 ± 0.20) was much higher than that of control subjects (1.16 ± 0.22, *p* < 0.001). Power calculation showed that our study had great power (100%) to detect the significant association of *PTBP1* methylation based on the nominal type I error rate of 0.01.

**TABLE 2:** DNA methylation difference of six CpG dinucleotides comparison within the *PTBP1* between IAs and Controls.

*PTBP1* methylation	Controls (*n* = 48)	IAs (*n* = 48)	t	*p*
CpG1 methylation (%)	1.89 ± 0.31	3.27 ± 0.26	3.428	**0.001**
CpG2 methylation (%)	2.59 ± 0.36	4.32 ± 0.28	3.78	**<0.001**
CpG3 methylation (%)	1.20 ± 0.48	2.22 ± 0.21	1.93	**0.037**
CpG4 methylation (%)	0.74 ± 0.22	1.72 ± 0.24	3.06	**0.003**
CpG5 methylation (%)	0.22 ± 0.16	1.94 ± 0.59	2.81	**0.006**
CpG6 methylation (%)	0.32 ± 0.18	1.37 ± 0.40	2.38	**0.020**
Mean methylation (%)*	1.16 ± 0.22	2.48 ± 0.20	4.46	**<0.001**

*Power = 100%, based on the nominal type I error rate of 0.01. p < 0.05 is bold.

**FIGURE 2:**
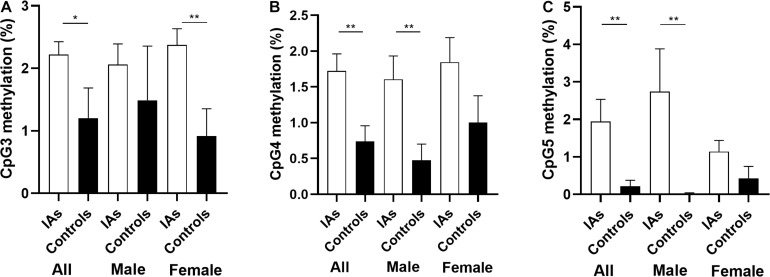
Comparison of *PTBP1* promoter CpG3, CpG4, CpG5 methylation between IAs and controls in different sex. **(A)** The comparison of CpG3 methylation among groups. **(B)** The comparison of CpG4 methylation among groups. **(C)** The comparison of CpG5 methylation among groups. *p < 0.05, **p < 0.001.

**FIGURE 3:**
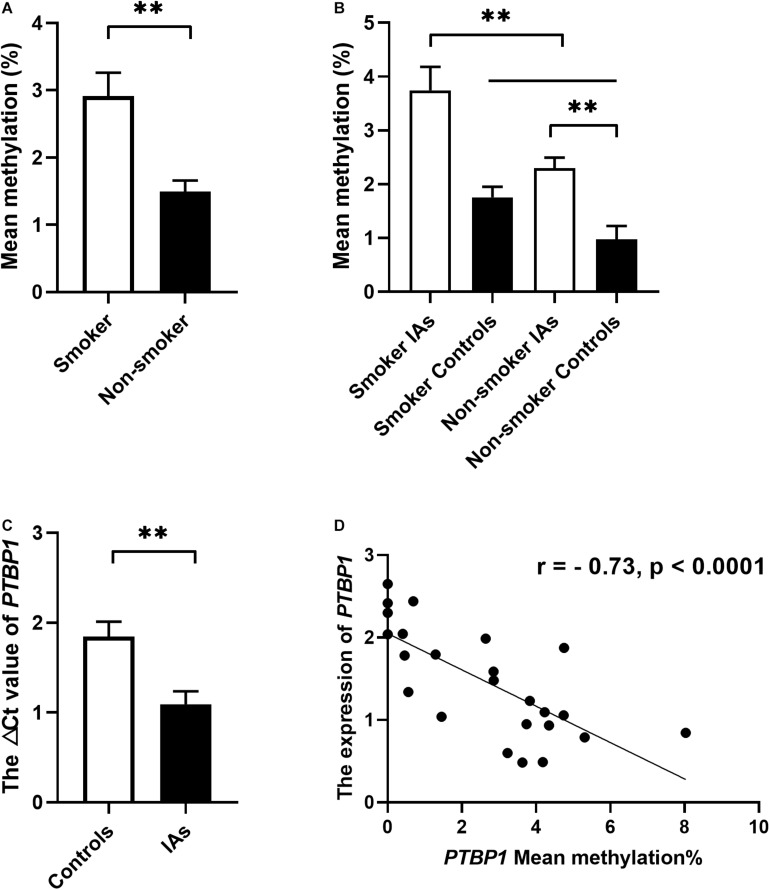
The relationship between *PTBP1* promoter DNA methylation, smoking, and gene expression in the individuals. **(A)** *PTBP1* DNA methylation was much higher in smokers than in non-smokers. **(B)** *PTBP1* DNA methylation was much higher in smoker IAs than other groups. **(C)** *PTBP1* mRNA expression was much lower in IAs than in healthy controls. **(D)** The *PTBP1* expression was significantly associated with DNA methylation in all individuals. **p < 0.001.

In addition, stratification analysis by sex showed that the mean methylation levels of *PTBP1* in both male (2.57 ± 0.34 vs. 1.09 ± 0.22, *p* = 0.001) and female (2.39 ± 0.21 vs. 1.23 ± 0.39, *p* = 0.011, Table 3) patients with IA were higher than the control group. However, the CpG3 methylation was significantly higher in female IA patients (IAs vs. Controls: 2.37 ± 0.26 vs. 0.92 ± 0.44, *p* = 0.006), but not in males (IAs vs. Controls: 2.06 ± 0.33 vs. 1.49 ± 0.87, *p* = 0.542, Figure 2A). The CpG4 and CpG5 methylation levels were significant higher in male IA patients (IAs vs. Controls: CpG4, 1.61 ± 0.32 vs. 0.47 ± 0.32, *p* = 0.007; CpG5, 2.74 ± 1.14 vs. 0.01 ± 0.00, *p* = 0.024), but not in females (IAs vs. Controls: CpG4, 1.84 ± 0.35 vs. 1.00 ± 0.37, *p* = 0.105; CpG5, 1.14 ± 0.30 vs. 0.43 ± 0.32, *p* = 0.108, Figures 2B,C). We also performed subgroup analysis stratified by smoking and non-smoking in all participants. Tobacco smokers had significantly higher levels of DNA methylation in *PTBP1* than non-smokers (2.92 ± 0.35 vs. 1.49 ± 0.17, *p* = 0.002, Figure 3A). The mean methylation levels of *PTBP1* in smoke IAs (3.74 ± 0.44) were significantly higher than that in smoke controls (1.76 ± 0.20, *p* = 0.005, Figure 3B), non-smoking IAs (2.29 ± 0.19, *p* = 0.007, Figure 3B), and non-smoker controls (0.97 ± 0.25, *p* < 0.001, Figure 3B). A similar result was also shown between non-smoker IAs and non-smoker controls (*p* = 0.002, Figure 3B). The *PTBP1* mRNA levels in the blood were much lower in IAs compared with controls (*p* = 0.002, Figure 3C). Moreover, the mRNA expression of *PTBP1* was significantly associated with the level of DNA methylation promotion in individuals (r = −0.73, *p* < 0.0001, Figure 3D). However, the following subgroup analysis stratified by ruptured and unruptured aneurysms in patients revealed no statistical difference in *PTBP1* methylation between ruptured IA and unruptured IA groups (Table 4, *p* > 0.05).

**TABLE 3:** Comparison of *PTBP1* methylation between two groups according to gender.

Characteristics	Controls (*n* = 24)	IAs (*n* = 24)	t	*P*
**Female**				
CpG1 methylation (%)	1.98 ± 0.54	3.46 ± 0.27	2.46	**0.019**
CpG2 methylation (%)	2.70 ± 0.62	4.64 ± 0.29	2.84	**0.008**
CpG3 methylation (%)	0.92 ± 0.44	2.37 ± 0.26	2.87	**0.006**
CpG4 methylation (%)	1.00 ± 0.37	1.84 ± 0.35	1.65	0.105
CpG5 methylation (%)	0.43 ± 0.32	1.14 ± 0.30	1.64	0.108
CpG6 methylation (%)	0.35 ± 0.24	0.84 ± 0.23	1.47	0.149
Mean methylation (%)	1.23 ± 0.39	2.39 ± 0.21	2.64	**0.011**
**Male**				
CpG1 methylation (%)	1.80 ± 0.33	3.08 ± 0.44	2.35	**0.023**
CpG2 methylation (%)	2.48 ± 0.39	4.00 ± 0.47	2.48	**0.017**
CpG3 methylation (%)	1.49 ± 0.87	2.06 ± 0.33	0.615	0.542
CpG4 methylation (%)	0.47 ± 0.23	1.61 ± 0.32	2.84	**0.007**
CpG5 methylation (%)	0.01 ± 0.00	2.74 ± 1.14	2.41	**0.024**
CpG6 methylation (%)	0.29 ± 0.29	1.91 ± 0.77	1.99	0.056
Mean methylation (%)	1.09 ± 0.22	2.57 ± 0.34	3.65	**0.001**

*p < 0.05 is bold.*

**TABLE 4:** Characteristics of ruptured IA and unruptured IA.

*PTBP1* methylation	Ruptured IA (*n* = 24)	Unruptured IA (*n* = 24)	t	*p*
CpG1 methylation (%)	3.43 ± 0.34	3.11 ± 0.38	0.63	0.535
CpG2 methylation (%)	4.71 ± 0.37	3.94 ± 0.40	1.40	0.168
CpG3 methylation (%)	2.26 ± 0.31	2.18 ± 0.28	0.19	0.853
CpG4 methylation (%)	1.87 ± 0.39	1.58 ± 0.27	0.63	0.533
CpG5 methylation (%)	2.32 ± 1.10	1.56 ± 0.46	0.64	0.528
CpG6 methylation (%)	1.73 ± 0.76	1.01 ± 0.26	0.89	0.376
Mean methylation (%)	2.72 ± 0.29	2.23 ± 0.26	1.25	0.219

**FIGURE 4:**
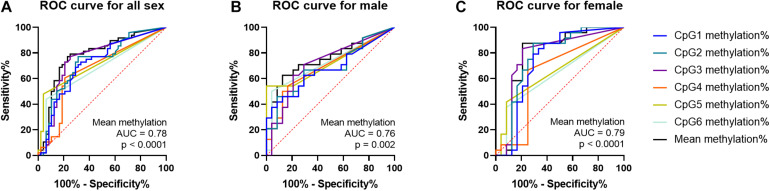
ROC curves of *PTBP1* DNA methylation in all sexes, male and female samples. **(A)** ROC curve for *PTBP1* DNA methylation in all sexes. **(B)** ROC curve for *PTBP1* DNA methylation in males. **(C)** ROC curve for *PTBP1* DNA methylation in females.

The ROC analyses of curves revealed that *PTBP1* methylation may be a predictor of IA regardless of sex (both sex, area under curve (AUC) = 0.78, *p* < 0.0001; male, AUC = 0.76, *p* = 0.002; female, AUC = 0.79, *p* < 0.0001, Figure 4).

## Discussion

IA formation was reportedly affected by the complex interaction between genetic and environmental risk factors (Vlak et al., 2013). The goal of this study was to explore the relationship between *PTBP1* gene methylation and the risk of IA in the Chinese Han population. Our results found that tobacco smoking could increase the level of *PTBP1* DNA methylation. The *PTBP1* methylation levels in IA patients were much higher than those found in healthy controls, whereas blood *PTBP1* expression in IAs was much lower than controls. The *PTBP1* methylation in CpG3, CpG4, and CpG5 showed a sex-dependent effect between IAs and controls.

*PTBP1* is widely expressed in the vasculature, including endothelial cells, VSMCs, and adventitial fibroblasts, and is involved in the pathophysiological changes of blood vessels (Caruso et al., 2017; Zhang et al., 2017). *PTBP1* regulates VSMC proliferation and neointimal hyperplasia during the development of vascular restenosis (Wang et al., 2019). *PTBP1* inhibition could attenuate the VSMC proliferation and suppress neointimal hyperplasia after vascular injury (Wang et al., 2019). Current studies believe that the main feature of IA pathogenesis is derived from the decreased extracellular matrix and the number of VSMCs in the cerebral vessel wall (Miyata et al., 2020). DNA methylation in the promoter region negatively correlates with gene expression, and is involved in the pathological process of cerebrovascular disease (Deng et al., 2019). Abnormal methylation of gene promoter participated in the regulation of VSMC (Han et al., 2014) proliferation and intimal hyperplasia (Wang et al., 2021) after vascular injury by affecting the expression of vascular-related genes. Such as, VSMCs mitochondrial D-loop methylation can lead to impaired mitochondrial function and loss of contractile phenotype in vascular disease (Liu et al., 2020). In the current study, our results showed that blood *PTBP1* levels in IA patients were much lower than that of healthy controls, which correlated with the higher level of DNA methylation in the *PTBP1* promotor region in IAs compared to controls. However, no significant difference was found in the methylation level of the *PTBP1* gene promoter region between ruptured and unruptured IAs. Therefore, DNA methylation of the *PTBP1* promotor may participate in the pathophysiological formation of IA by attenuating *PTBP1* expression in patients.

Long-term heavy smoking abuse is an important factor for epigenetic modifications, including DNA methylation in human diseases (Hillemacher et al., 2019; Lee and Park, 2020). Cigarette smoke can promote cerebrovascular disease in an important pathway by inducing the inflammatory/matrix remodeling phenotype in cerebral VSMCs (Starke et al., 2013). It was also suggested that smoking tobacco affects the regulation of gene expression by affecting DNA methylation, thereby increasing the risk of cardiovascular disease (Haase et al., 2018; Siemelink et al., 2018). Studies have shown that long-term smoking could increase or decrease the level of DNA methylation in the promoter region of genes, further alter the level of gene expression, and participate in the pathological process of smoking-related diseases (Huan et al., 2016). Our previous study proved that regular tobacco smoking could increase the methylation of nitric oxide synthase 1 adaptor protein, and could increase the risk of IA and cerebral arteriovenous malformations in Han Chinese (Wang et al., 2016). In the current study, our results showed that smoking could increase the level of *PTBP1* promotor methylation in Han Chinese individuals. Therefore, tobacco smoking may be involved in the pathophysiological formation of IA by increasing the level of DNA methylation in the *PTBP1* promotor and by attenuating *PTBP1* expression in the patients.

Sex-specific differences have been reported in IA outcomes (Vlak et al., 2011) and many gene DNA methylation rates (Bain et al., 2021). Compared with males, the females had a much incidence of unruptured aneurysms, as well as a much higher risk of rupture (Yanagisawa et al., 2020). Besides, males and females will also have different methylation reactions to changes in the external environment. For example, when people are exposed to polybrominated biphenyls (Curtis et al., 2020) or perinatally exposed to lead (Wang et al., 2020), they can induce tissue- and sex-specific DNA methylation changes. In the present study, *PTBP1* CpG3 methylation levels were much higher in female IAs than in the controls, but higher levels of CpG4 and CpG5 methylation were found in male IAs. However, the average methylation of *PTBP1* promotor between the case and control groups is consistent in male and female groups. Moreover, The ROC analyses revealed that *PTBP1* methylation may be a predictor of IA regardless of sex.

## Study Limitations

Some limitations remain in our work. Firstly, although our study has excellent statistical power, the sample size was relatively small, and more sample tests, including various ages of smokers, will be needed to confirm our findings in future studies. Secondly, only six GpG dinucleotides on a fragment (chr19:796,392–797,191) were selected to represent the entire promoter of the *PTBP1* gene. More CpGs should be included in future studies. Thirdly, this was a candidate study, but the mechanism research was likely not as thorough as it should have been. Animal models and cell experiments are necessary to further verify and validate future results.

## Conclusion

Collectively, these findings suggest that long-term tobacco smoke exposure led to DNA methylation in the promoter region of the *PTBP1* gene, which further decreased *PTBP1* gene expression, and participated in the pathogenesis of IA. *PTBP1* methylation may be a potential predictive marker for the occurrence of IA.

## Data Availability Statement

The original contributions presented in the study are included in the article/supplementary material, further inquiries can be directed to the corresponding author/s.

## Ethics Statement

The studies involving human participants were reviewed and approved by the Ethics Committee of Ningbo First Hospital.

## Author Contributions

YH, ZL, and GC contributed to the conception and design of the study. ZW, SZ, JZ, SN, JS, and XG organized the database and experiments. SZ and YH performed the statistical analysis. ZW and YH wrote the first draft of the manuscript. YH and CL wrote sections of the manuscript. All authors contributed to manuscript revision, read, and approved the submitted version.

## Conflict of Interest

The authors declare that the research was conducted in the absence of any commercial or financial relationships that could be construed as a potential conflict of interest.
